# Honokiol inhibits sphere formation and xenograft growth of oral cancer side population cells accompanied with JAK/STAT signaling pathway suppression and apoptosis induction

**DOI:** 10.1186/s12885-016-2265-6

**Published:** 2016-03-24

**Authors:** Jhy-Shrian Huang, Chih-Jung Yao, Shuang-En Chuang, Chi-Tai Yeh, Liang-Ming Lee, Ruei-Ming Chen, Wan-Ju Chao, Jacqueline Whang-Peng, Gi-Ming Lai

**Affiliations:** Comprehensive Cancer Center, Taipei Medical University, Taipei, Taiwan; Cancer Center, Wan Fang Hospital, Taipei Medical University, No.111, Section 3, Hsing-Long Road, Taipei, 116 Taiwan; Department of Internal Medicine, School of Medicine, College of Medicine, Taipei Medical University, No.250, Wuxing Street, Taipei, 110 Taiwan; National Institute of Cancer Research, National Health Research Institutes, Miaoli County, Taiwan; Department of Surgery, Shuang Ho Hospital, Taipei Medical University, Taipei, Taiwan; Department of Urology, School of Medicine, College of Medicine, Taipei Medical University, Taipei, Taiwan; Graduate Institute of Medical Sciences, College of Medicine, Taipei Medical University, Taipei, Taiwan

**Keywords:** Honokiol, Cancer stem-like side population, JAK2/STAT3 pathway, Oral cancer

## Abstract

**Background:**

Eliminating cancer stem cells (CSCs) has been suggested for prevention of tumor recurrence and metastasis. Honokiol, an active compound of *Magnolia officinalis*, had been proposed to be a potential candidate drug for cancer treatment. We explored its effects on the elimination of oral CSCs both in vitro and in vivo.

**Methods:**

By using the Hoechst side population (SP) technique, CSCs-like SP cells were isolated from human oral squamous cell carcinoma (OSCC) cell lines, SAS and OECM-1. Effects of honokiol on the apoptosis and signaling pathways of SP-derived spheres were examined by Annexin V/Propidium iodide staining and Western blotting, respectively. The in vivo effectiveness was examined by xenograft mouse model and immunohistochemical tissue staining.

**Results:**

The SP cells possessed higher stemness marker expression (*ABCG2*, *Ep-CAM*, *Oct-4* and *Nestin*), clonogenicity, sphere formation capacity as well as tumorigenicity when compared to the parental cells. Treatment of these SP-derived spheres with honokiol resulted in apoptosis induction via Bax/Bcl-2 and caspase-3-dependent pathway. This apoptosis induction was associated with marked suppression of JAK2/STAT3, Akt and Erk signaling pathways in honokiol-treated SAS spheres. Consistent with its effect on JAK2/STAT3 suppression, honokiol also markedly inhibited IL-6-mediated migration of SAS cells. Accordingly, honokiol dose-dependently inhibited the growth of SAS SP xenograft and markedly reduced the immunohistochemical staining of PCNA and endothelial marker CD31 in the xenograft tumor.

**Conclusions:**

Honokiol suppressed the sphere formation and xenograft growth of oral CSC-like cells in association with apoptosis induction and inhibition of survival/proliferation signaling pathways as well as angiogenesis. These results suggest its potential as an integrative medicine for combating oral cancer through targeting on CSCs.

**Electronic supplementary material:**

The online version of this article (doi:10.1186/s12885-016-2265-6) contains supplementary material, which is available to authorized users.

## Background

Oral squamous cell carcinoma (OSCC) is the most common type of head and neck cancer, which is estimated over 200,000 new cases and 120,000 deaths worldwide [1]. In Taiwan, OSCC has emerged as one of the major malignancies with high increasing rate of both incidence and mortality in the past decade [2]. First-line combination chemotherapy with docetaxel, cisplatin and 5-flurouracil (TPF) nowadays has been the most commonly used induction regimen for the treatment of advanced diseases (stages III and IV), but the side effects are severer than single-drug chemotherapy [3, 4]. Despite the improvements of surgical and radiation techniques, the 5-year survival rate of oral cancer has remained unchanged at about 50 % over the past 30 years [5]. Local recurrence and distant metastases are two critical influencing factors on survival of OSCC. Therefore, it is urgent to develop more effective agents for the improvement of clinical outcome.

According to the model of cancer stem cells (CSCs), increasing evidence suggests that tumor recurrence and metastases are caused exclusively by a rare subpopulation of tumor-initiating cells with stem cell properties [6–9]. CSCs exhibit capacities of self-renewal, tumorigenicity and differentiating into non-stem cancer cells that constitute the bulk of tumors [10, 11]. Thus, targeting the CSCs population has become a novel strategy to prevent tumor recurrence or metastasis. How to eradicate the existing CSCs to improve the survival of patients with OSCC after surgery and radio- or chemo-therapy becomes a challenging issue.

Isolation of CSCs from solid tumors has been successfully achieved through several methods based on the properties of CSCs [7, 12]. One common method is the side population (SP) technique based on the ability of these cells to efflux a fluorescent DNA-binding dye Hoechst 33342, as first described by Goodell [13]. The SP cells are a subset of cells harboring stem cell-like properties that show a distinct low Hoechst 33342 dye staining pattern [14]. Some studies demonstrated that SP cells isolated from various cancer cell lines showed high expression of stemness markers and the ability to initiate tumor formation as well as resistance to chemotherapy [14, 15]. Thus, it is postulated that SP cells are enriched of CSCs and represent an important potential target for novel anticancer drug development. Several reports had shown that SP cells possessing properties of CSCs could be isolated from OSCC cell lines [16–18], however, little is known about the eradication of these CSCs. Based on our previous studies, natural products and phytochemicals are the potential source of CSC targeting agents [19–22].

Honokiol is a bioactive compound purified from the bark of traditional Chinese herbal medicine *Magnolia* species. Evidences from in vitro and animal models had demonstrated that honokiol possessed a variety of pharmacological effects, such as anti-inflammation, anti-angiogenesis, anti-arrhythmic and antioxidant activity [23, 24]. It had also been shown to exert various protecting effects against hepatotoxicity, neurotoxicity, thrombosis and angiopathy [23]. The anticancer activity of honokiol had been demonstrated in a variety of cancer cell lines, including breast, lung, ovary, prostate, gastrointestinal and oral cancer cells as well as in xenograft animal models [24–26]. Our previous work and the study by Ponnurangam et al. had demonstrated the eliminating effect of honokiol on the CSCs-like population in OSCC and colon cancer cells through inhibition of Wnt/β-catenin [20] and Notch [27] pathway, respectively. In addition to the above stemness-associated pathways, several well-known survival/proliferation pathways such as JAK/STAT [28], PI3K/Akt [29, 30] and MEK/Erk [30, 31] had been shown to govern the maintenance and survival of CSCs. However, the effects of honokiol on these pathways of CSC are remained to be elucidated. Hence, it is interesting and worth to investigate honokiol-mediated elimination of CSCs in association with inhibition of these pathways.

In this study, we investigated honokiol-mediated suppression on these survival/proliferation signaling pathways in CSCs-enriched SP from OSCC cells and examined the in vivo effectiveness by xenograft mouse model and immunohistochemical tissue staining. As expected, our results showed that honokiol inhibited these pathways in SP spheres from SAS oral cancer cells and reduced the growth and immunohistochemical staining of xenograft tumor.

## Methods

### Cell lines and sphere culture

Eight human oral squamous cell carcinoma (OSCC) cell lines (FaDu, KB, OE, OECM-1, SAS, SCC4, SCC25 and YD10B) were maintained in RPMI 1640 with 10 % FBS and 1 % penicillin/streptomycin at 37^0^C, 5 % CO_2_, in a humidified chamber. After sorting, the side population cells were seeded at a density of 500 cells/well in 6-well ultra-low attachment plates (Corning Life Science, Corning, NY, USA) with HEscGro medium (Millipore, Billerica, MA, USA) containing epidermal growth factor (EGF, 10 ng/ml) plus basic fibroblast growth factor (bFGF, 8 ng/ml) but without any serum. The spheres were harvested after 14 days of culture for subsequent assays. The non-SP cells were incubated with serum-containing RPMI medium.

### Chemicals and reagents

Honokiol (purity >98 %) was kindly provided by Dr. Jack L. Arbiser, Emory University, USA. It was dissolved in dimethyl sulfoxide (DMSO) and further diluted in sterile culture medium for in vitro experiments. The final concentrations of DMSO in cell cultures were all less than 0.05 %. The antibodies against Bax (B-9, mouse monoclonal antibody, sc-7480), Bcl-2 (100, mouse monoclonal antibody, sc-509), Erk (K-23, rabbit polyclonal antibody, sc-94), phospho-Erk (E-4, mouse monoclonal antibody, sc-7383) and STAT3 (F-2, mouse monoclonal antibody, sc-8019) were purchased from Santa Cruz Biotechnology Inc. (Santa Cruz, CA, USA). The antibodies against caspase 3 (5A1E, rabbit monoclonal antibody, #9664), Akt (5G3, mouse monoclonal antibody, #2966), phospho-Akt (587 F-11, mouse monoclonal antibody, #4051), JAK2 (D2E12, rabbit monoclonal antibody, #3230), phospho-JAK2 (D4A8, rabbit monoclonal antibody, #8082) and phospho-STAT3 (D3A7, rabbit monoclonal antibody, #9145) were obtained from Cell Signaling Technology (Beverly, MA, USA).

### Identification and purification of side population

The side population (SP) cells were analyzed and sorted by Hoechst 33342 (Sigma) staining and FACSAria™ III sorter (BD Biosciences, San Jose, CA, USA). Cells were detached from dishes with Trypsin-EDTA (Invitrogen, Grand Island, NY, USA) and suspended at 1 × 10^6^ cells/mL in Hanks balanced salt solution (HBSS) supplemented with 3 % fetal calf serum and 10 mM HEPES. These cells were then incubated at 37 °C for 90 min with 2.5 μg/mL Hoechst 33342, either alone or in the presence of 50 μM reserpine (Sigma), a nonspecific inhibitor of drug-resistance ATP-binding cassette (ABC) pumps. The diminishment of SP cells in the presence of reserpine was used to define the flow cytometry gate for sorting SP cells. After 90-minute incubation, the cells were centrifuged for 5 min at 300 x *g*, 4 °C and resuspended in ice-cold HBSS. The cells were kept on the ice to inhibit efflux of Hoechst dye and 1 μg/mL propidium iodide (BD) was then added to discriminate dead cells. Finally, these cells were filtered through a 40 μm cells trainer (BD) to obtain single suspension cells for the analysis and sorting on FACSAria III flow cytometer.

### In vivo tumorigenicity assay

Dispersed cells were re-suspended in PBS. A 100 μL suspension containing various numbers of SP or non-SP cells were injected subcutaneously into the right flanks of 4- to 5-week-old male NOD/SCID mice, obtained from Taiwan University Animal Center (Taipei, Taiwan). The animal study protocols were approved by the institutional animal care and use committee of National Heath Research Institutes, Taiwan. Tumor volume was measured on a weekly basis by a digital caliper and calculated using the following formula: 0.52 × L × W^2^ (L, longest diameter; W, shortest diameter). The experiment was terminated 10 weeks after tumor cells inoculation and mice were euthanized. The tumor’s wet weight was then measured.

### Sphere formation assay

The spheres were collected by gentle centrifugation, dissociated with trypsin-EDTA and then mechanically pipetted. The resulting single cells were re-centrifuged to remove trypsin-EDTA and re-suspended in SP medium to allow spheres re-formation. The spheres were passaged every 5–7 days before they reached a diameter of 100 μm. For the sphere formation assay, the SP and non-SP cells were seeded at a low density of 20 cells/μL in the SP medium as described above. Ten days after plating, the number of spheres (>50 μm) formed was counted under a microscope.

### Colony formation assay

Cells were plated at a density of 500 cells/well on 6-well plates and cultured in serum-containing RPMI media at 37 °C in 5 % CO_2_ for 2 weeks. The number of colonies was counted after crystal violet staining (Sigma).

### Reverse transcription polymerase chain reaction (RT-PCR)

Trizol reagent was used to extract the mRNAs from the SAS SP and parental cells according to the manufacturer’s recommended protocol. Two μg RNA was added to RT-PCR reactions containing primers at a concentration of 0.5 μM. After a 42 °C/60-min reverse transcription step, 25–36 cycles of PCR amplification were performed at 94 °C for 30 s, 55 °C for 50 s, and 72 °C for 50 s. PCR products were run on 1.5 % agarose gels for identification. Primers used were, for *ABCG2*, forward: 5′-CATCAACTTTCCGGGGGTGA-3′ and reverse: 5′-TGTGAGATTGACCAACAGACCA-3′; for *EpCAM*, forward: 5′-CTGCCAAATGTTTGGTGATG -3′ and reverse: 5′-ACGCGTTGTGATCTCCTTCT-3′; for *Oct-4*, forward: 5′-GGAGAGCAACTCCGATGG-3′ and reverse: 5′-TTGATGTCCTGGGACTCCTC-3′; for *Nestin*, forward: 5′-CTCTGACCTGTCAGAAGAAT-3′ and reverse: 5′-GACGCTGACACTTACAGAAT-3′; for *GAPDH*, forward: 5′-ACCACAGTCCATGCCATCAC-3′ and reverse: 5′-TCCACCACCCTGTTGCTGTA-3′.

### Apoptosis analysis by Annexin V and Propidium iodide (PI) double staining

The Annexin V-FITC Apoptosis Detection Kit (BD Biosciences, San Jose, CA, USA) was used. In brief, the harvested cells were re-suspended in 1x binding buffer at a density of 1 × 10^6^ cells/mL and cells of each 100 μl aliquot were stained with Annexin V-PI labeling solution (containing 5 μl Annexin V-FITC and 5 μl propidium iodide) at room temperature in the dark for 15 min. Finally, binding buffer (400 μl) was added and the cells were analyzed by flow cytometer.

### Western blot analysis

The SP-derived spheres were collected and lysed in RIPA buffer containing protease inhibitors. Protein concentrations were measured by using the BCA protein assay kit (Thermo Scientific Biosciences, Rockford, IL, USA). Quantified protein lysates were separated by SDS-PAGE, transferred onto PVDF membrane (Millipore, Billerica, MA, USA) and immunoblotted with the primary antibodies. After incubation with HRP-conjugated secondary antibody, immunoreactive bands were visualized by enhanced chemiluminescence detection system (Millipore, Billerica, MA USA). The protein bands were quantified by AlphaEaseFC™ software.

### Knockdown of STAT3

STAT3 siRNA was purchased from Cell Signaling (SignalSilence® Stat3 siRNA II #6582). The mismatch siRNA oligonucleotide 5′-UCGGCUCUUACGCAUUCAA-3′ was used as a siRNA control. Cells were transfected with siRNA oligonucleotide using Oligofectamine reagent according to the manufacturer’s instructions (Invitrogen, Grand Island, NY, USA) and analyzed 72 h post-transfection.

### Wound healing assay

SAS cells were seeded into a 6-well plate. After growing to confluence, straight scratches were made across the monolayer by using a white tip along plate cover. Then, IL-6 (50 ng/ml) or honokiol (5 μM) was added into wells as indicated and recorded by photography 24 h later.

### Xenograft assay

NOD/SCID mice were inoculated subcutaneously with 5 × 10^3^ SAS SP cells into the flank and allowed to grow. Mice were randomly divided into four groups (*n* = 5): vehicle control (1 % carboxymethyl cellulose, CMC, Sigma) and honokiol-treated groups at different dose (20, 40, 80 mg/kg). Three weeks after inoculation, honokiol (diluted in 1 % CMC immediately prior to administration) was given intraperitoneally to mice thrice a week until week 10. At the end, mice were sacrificed and the tumors were paraffin embedded for the immunohistochemical staining of PCNA (PC10, mouse monoclonal antibody, #2586, Cell Signaling Technology, Beverly, USA) and CD31 (JC/70A, mouse monoclonal antibody, ab9498, Abcam, Cambridge, UK). The PCNA labeling index was calculated as the percentage of positively stained nuclei in a total of 600 cells in 3 different areas. The vascular density was determined by counting the number of CD31-positive microvessels per high-power field (x200) [32].

### Statistical analysis

Quantitative data were shown as mean ± SD. Differences between control and honokiol-treated groups were analyzed by Student’s *t*-test. A *p*-value of <0.05 was considered statistically significant in each experiment.

## Results

### Identification of SP cells in OSCC cell lines

**Fig. 1:**
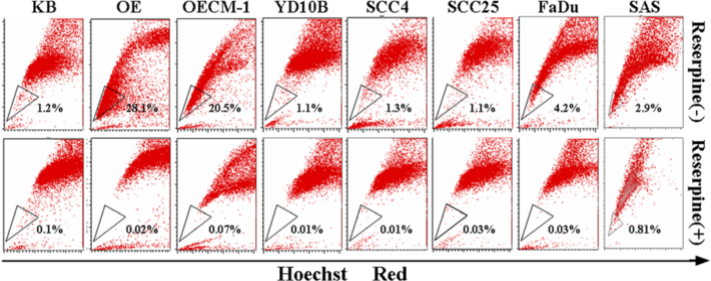
Percentage of side population cells in oral squamous cell carcinoma cells lines. Eight human oral squamous cell carcinoma cell lines were stained with Hoechst 33342 dye in the presence (*bottom*) or absence (*upper*) of 50 μM reserpine and analyzed by flow cytometry. The side population cells (*black triangle*), which were disappeared by reserpine, are shown as a percentage of the whole living cell population

We examined the existence of SP cells in eight human OSCC cell lines by staining with Hoechst 33342 dye to generate a Hoechst blue-red profile. In each cell line, the percentage of SP cells was markedly diminished by treatment with reserpine, which is an inhibitor of the ABC pumps responsible for the exclusion of Hoechst dye, indicating that this population truly represented SP cells. As depicted in Fig. 1, all the OSCC cell lines contained a distinct fraction of SP cells, ranging from 1.1 % (YD10B and SCC25) to 28.1 % (OE) of gated cells.

### Side population-derived sphere cells have stem cell properties

**Fig. 2:**
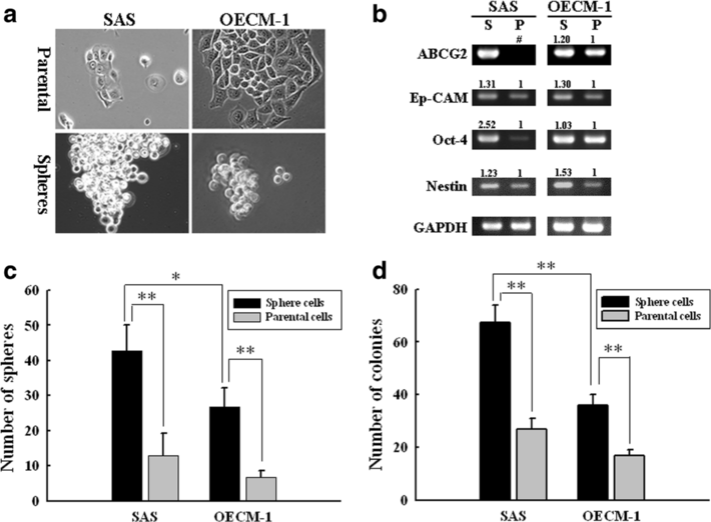
SP-derived spheres from SAS and OECM-1 cell lines possess the stemness properties. **a** After cultured in an anchorage-independent manner for 7 days, the spheroidal morphology (phase-contrast images) of SAS (*left*) and OECM-1 (*right*) sphere cells were distinct from those of parental cells. **b** Marked higher expression of stemness markers in SAS and OECM-1 sphere (“S”) cells compared to parental (“P”) cells. The expression of various stemness markers was analyzed by RT-PCR and GAPDH was used as a loading control. The intensities of the PCR bands were quantified by densitometry. The densitometric values indicated at the top of the bands are expressed relative to the value of parental cells after being normalized to actin (#: The intensity of ABCG2 band of parental SAS cells was undetectable in this PCR condition). Both the SAS and OECM-1 sphere cells had higher capacities in sphere (**c**) and colony (**d**) formation than parental cells. Data are shown as mean ± SD from experiments performed in triplicates. *, *p* < 0.05; **, *p* < 0.01

To investigate the CSCs of OSCC cells with different aggressiveness, SP cells from SAS (high malignancy and metastasis) and OECM-1 (less malignancy) [33] were chosen and cultured to form spheres according to the methods described. The spheres derived from SAS and OECM-1 SP cells appeared to be taking shape on day 4 and were completely formed on day 10. Morphologically, these spheres grew tightly in clusters in three-dimensional configuration in contrast to the flattened shape of parental cells (Fig. 2a). We then examined the expression of stemness markers in these SP-derived spheres and parental cells by RT-PCR. As shown in Fig. 2b, the mRNA expressions of *ABCG2*, *Ep-CAM*, *OCT-4* (octamer-binding transcription factor 4) and *Nestin* was higher in sphere cells than those in their parental cells. These SP cells also possessed higher self-renewal ability as they formed much higher number of spheres in the serum-free SP medium (Fig. 2c). In parallel with this, the SP cells formed markedly higher number and larger size of colonies than the parental cells in serum-containing culture medium (Fig. 2d).

Comparing the stemness properties of SAS and OECM-1 sphere cells, we found the elevation of *ABCG2* and *Oct-4* expressions in SAS spheres were much more marked than that in OECM-1 spheres (Fig. 2b). This result indicated that the SAS sphere cells were more CSCs-like than those of OECM-1. Besides, the SAS SP cells also possessed higher capability of sphere and colony formation than the OECM-1 SP cells (Fig. 2c and d). As these stemness characteristics found in vitro are considered to render the tumorigenicity of SP cells in vivo, our findings are in consistent with the report by Chang et al. that SAS cells are much more tumorigenic than OECM-1 cells [33, 34].

### SAS SP cells show more tumorigenic potential in xenografts

**Fig. 3:**
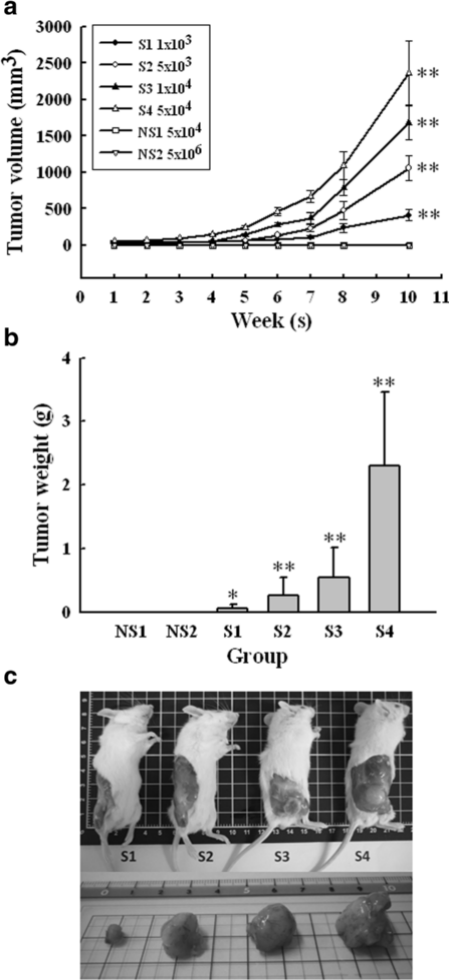
Side population cells (S1-S4) possess higher tumorigenicity than non-side population cells (NS1-NS2). **a** The growth curve of xenograft tumor. NOD/SCID mice were inoculated subcutaneously with various cell numbers of SAS SP and non-SP cells, respectively, as indicated. Tumor volumes were recorded on a weekly basis. **p < 0.01, significant difference vs. NS1. **b** The wet weight of tumors measured after harvested at the end. *p < 0.05; **p < 0.01, significant difference vs. NS1. **c** Representative photographs of the tumors harvested at the end of experiment

To further characterize the stemness properties of SAS SP cells, we examined the tumorigenicity of SAS SP and non-SP cells in vivo. Various numbers of SP (S1-S4) and non-SP (NS1-NS2) cells were subcutaneously inoculated into NOD/CSID mice. As shown in Fig. 3a, the volume of SP cell-derived tumors increased in a cell number- and time-dependent manner. The SP cells formed tumors in three out of five mice, even the number of inoculated cells was as low as 1 × 10^3^ (Table 1). In addition, the tumor weights were also measured and found to be increased with the number of SP cells inoculated (Fig. 3b). In contrast, no tumors were formed in mice inoculated with non-SP cells, even the number of inoculated non-SP cells was up to 1 × 10^6^, and only two out of five mice formed tumors when 1 × 10^7^ non-SP cells were inoculated (Table 1). The photograph of representative sizes of SP cells-derived tumors in each group was shown in Fig. 3c. Based on our results, the tumorigenicity of SAS SP cells was estimated to be ten-thousand times higher than non-SP cells.

**Table 1:** Tumorigenicity of SAS SP and non-SP cells in NOD/SCID mice

	Cell numbers inoculated/mouse					
	1 × 10^3^	5 × 10^3^	1 × 10^4^	5 × 10^4^	1 × 10^6^	5 × 10^7^
SAS SP	3/5	3/5	4/5	5/5	—	—
SAS non-SP	—	—	—	0/5	0/5	2/5

The number of mice with tumor formation/total number of mice inoculated with SAS SP or non-SP cells was observed for 10 weeks after inoculation. —, not done

### Honokiol inhibits the colony formation and induces apoptosis in sphere cells

**Fig. 4:**
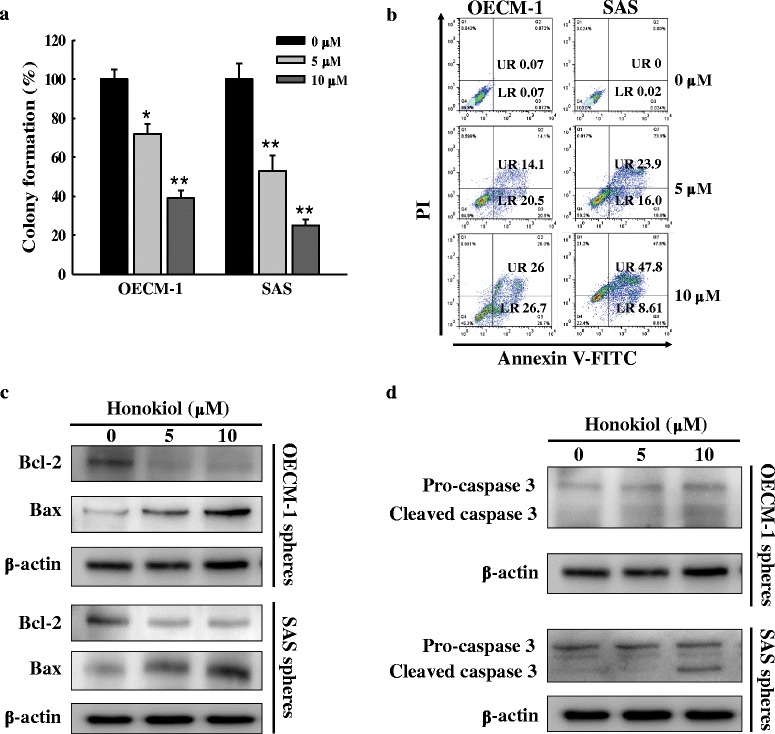
Honokiol inhibits colony formation and induces apoptosis via Bax/Bcl-2 and caspase-3-dependent pathway in SP-derived sphere cells. **a** Honokiol inhibited colony formation of the SAS and OECM-1 SP-derived sphere cells in a dose-dependent manner. The colony formation data are expressed as percent of control (without honokiol treatment) cells and shown as mean ± SD. *p < 0.05; **p < 0.01, significant difference vs. control. **b** Honokiol induced apoptosis of the SAS and OECM-1 SP-derived spheres in a dose-dependent manner. Apoptosis was determined by Annexin V-FITC/PI double staining and flow cytometry analysis. The honokiol concentration is shown in the *right side of dot plots*. The numbers in LR (*lower right*) quadrant indicates the percentage of early apoptotic cells. The numbers in UR (*upper right*) quadrant indicates the percentage of late apoptotic cells. **c** Dose-dependent effect of honokiol on the protein levels of Bax and Bcl-2. **d** Dose-dependent effect of honokiol on cleavage of caspase-3

To evaluate the effects of honokiol on the elimination of CSCs in OSCC, we examined its effects on the colony formation and apoptosis induction in these sphere cells. In OECM-1 sphere cells, the number of colonies was dose-dependently decreased to 70 and 38 % by honokiol at dose of 5 and 10 μM, respectively. In SAS sphere cells, the number of colonies was even down to 50 and 22 % by the same doses of honokiol (Fig. 4a). After 48 h of honokiol treatment, apoptosis was induced in both OECM-1 and SAS sphere cells in a dose-dependent manner (Fig. 4b). At a dose of 10 μM, honokiol-induced apoptosis was up to 52.7 and 56.41 % in OECM-1 and SAS sphere cells, respectively (Fig. 4b). Moreover, the honokiol-induced late apoptosis (upper-right quadrant) was more dominant in SAS sphere (23.9 and 47.8 %) than in OECM-1 sphere (14.1 and 26 %) cells (Fig. 4b). Taken together with the result shown in Fig. 4a, the higher malignant and tumorigenic SAS spheres appeared to be more sensitive to honokiol-induced anticancer effects than the OECM-1 sphere cells.

We then examined the changes in levels of the Bcl-2 and Bax proteins that regulate the intrinsic apoptosis pathway of cancer cells. Both in OECM-1 and SAS sphere cells, honokiol decreased the anti-apoptotic Bcl-2 while increased the pro-apoptotic Bax protein in a dose-dependent manner (Fig. 4c). As expected, this increase of Bax to Bcl-2 protein ratio led to cleavage/activation of the key apoptosis co-ordination enzyme, caspase-3, in both of the two cancer spheres (Fig. 4d). These results suggest the pivotal role of mitochondria-dependent (intrinsic) apoptosis in honokiol-mediated elimination of CSCs in OSCC cells.

### Honokiol inhibits the JAK2/STAT3, Akt and Erk signal pathways in SAS sphere cells

**Fig. 5:**
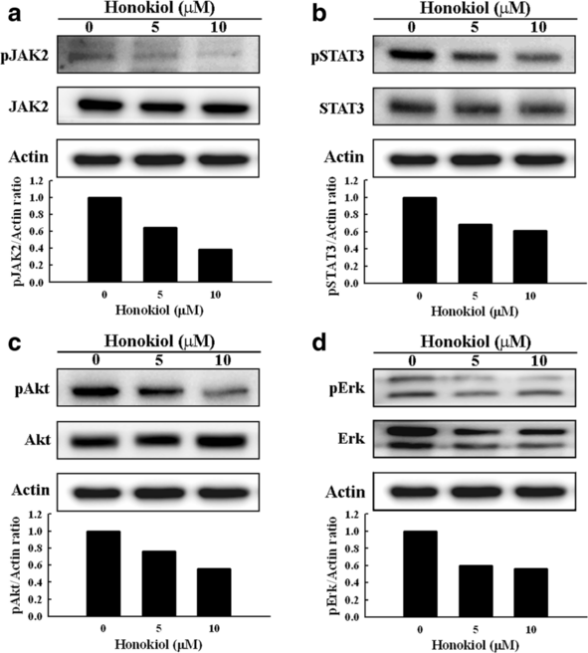
Honokiol inhibits the JAK2/STAT3, Akt and Erk pathways in SP-derived spheres. The SAS SP-derived spheres were incubated with 5 or 10 μM honokiol for 48 h. The protein levels of total and phosphorylated JAK2 (**a**), STAT3 (**b**), Akt (**c**) and Erk (**d**) were determined by Western blot and quantified by densitometry. The ratios of pJAK2, pSTAT3, pAkt and pErk to actin were calculated

Regarding the profound inhibition of colony formation and induction of apoptosis shown in Fig. 4, we examined the survival/proliferation signals such as JAK2/STAT3, Akt and Erk pathways in honokiol-treated SAS sphere cells. After 48 h of treatment, honokiol markedly decreased the levels of phospho-JAK2 (pJAK2) and phospho-STAT3 (pSTAT3) rather than affecting the total protein levels of JAK2 and STAT3 (Fig. 5a and b). Honokiol also dose-dependently decreased the phospho-Akt (pAkt) without affecting the total Akt protein level (Fig. 5c). Both the phospho-Erk (pErk) and total Erk were simultaneously reduced by honokiol (Fig. 5d). These survival/proliferation signaling pathways might be suppressed through different mechanisms during apoptosis induction by honokiol in the CSC-like sphere cells.

### Honokiol suppresses the migration of SAS cells

**Fig. 6:**
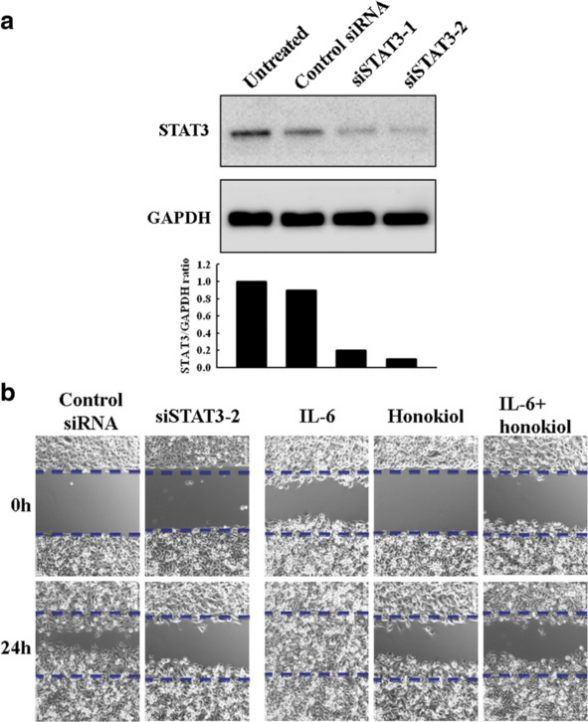
Honokiol suppresses IL-6-mediated migration of SAS cells. **a** Two preparations of SAS cells were transfected with STAT3 siRNA (siSTAT3-1, siSTAT3-2) and the control siRNA group was transfected with the mismatch siRNA oligonucleotide. After 72 h, the STAT3 expression was determined by Western blot. **b** Wound healing assay. The STAT3 siRNA transfected SAS cells (siSTAT3-2) were seeded into a 6-well plate. After growing to confluence, straight scratches were made across the monolayer by using a white tip along plate cover. Then, IL-6 (50 ng/ml) or honokiol (5 μM) was added into wells as indicated and recorded by photography 24 h later. Honokiol inhibited the migration of SAS cells as potent as STAT3 siRNA. Notably, honokiol also suppressed the migration enhanced by IL-6. In this assay, honokiol (5 μM) and siSTAT3 did not affect the cell viability of these cells (Additional file 1: Figure S1)

The JAK2/STAT3 pathway regulates not only the anti-apoptotic survival signal but also the motility of cancer cells [35]. Considering the marked JAK2/STAT3 pathway inhibition by honokiol, we explored its effect on cell migration (the wound healing assay) of the highly aggressive SAS cells, using STAT3 siRNA as a positive control. As shown in Fig. 6a, marked decrease of STAT3 protein expression was observed in the two preparations of STAT3 siRNA transfected cells (siSTAT3-1, siSTAT3-2). As expected, the migration of siSTAT3-2-transfected cells was significantly inhibited as compared to that of the cells transfected with control siRNA (Fig. 6b). In consistent with the inhibition on JAK2/STAT3 pathway shown in Fig. 5, honokiol inhibited SAS cell migration as effective as the siSTAT3 after 24 h of incubation (Fig. 6b). As the JAK2/STAT3 pathway in human malignancies could be triggered by the pro-inflammatory cytokine such as IL-6 [36], we further investigated the inhibitory effect of honokiol on IL-6-mediated cell migration. Notably, we found honokiol could suppress the migration enhanced by IL-6 as well (Fig. 6b).

### Honokiol inhibits the tumor growth of SAS SP xenograft

**Fig. 7:**
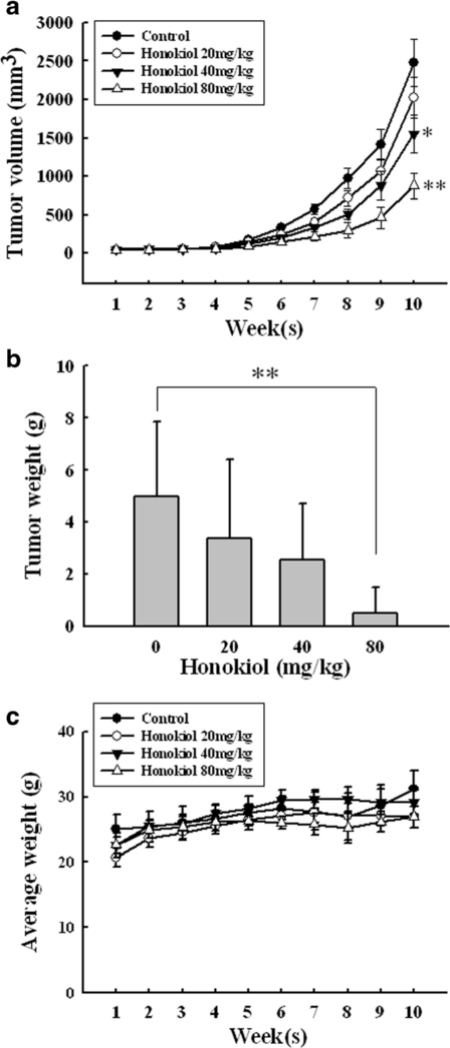
Honokiol dose-dependently inhibits growth of SAS SP cells xenograft in NOD/SCID mice. Mice were inoculated subcutaneously with 5 × 10^3^ SAS SP cells. Honokiol was administered by intraperitoneal injection thrice a week. **a** Tumor volumes were measured once a week. The tumor growth was dose-dependently inhibited by honokiol. *p < 0.05; **p < 0.01, significant difference vs. control. **b** At the end of week 10, the tumors were harvested and weighed. Honokiol dose-dependently decreased the tumor weight. Data shown are mean ± SD (*n* = 5). **p < 0.01, significant difference vs. control. **c** The changes of body weight were measured weekly after honokiol treatment. No significant difference between control and honokiol-treated groups was observed

To confirm the effectiveness in vivo, we examined the effects of honokiol on the tumor growth of SAS SP xenograft in SCID mice. The tumor volume was periodically measured with a metric caliper and the body weight was also simultaneously measured on a weekly basis. The tumor volume of control group gradually increased to 2479 ± 302 mm^3^ after subcutaneous inoculation with 5 × 10^3^ SAS SP cells for 10 weeks (Fig. 7a). At week 10, honokiol decreased the tumor volume to 2024 ± 265, 1555 ± 247 and 879 ± 166 mm^3^ at doses of 20, 40 and 80 mg/kg, respectively (Fig. 7a). By calculation, the percentage of tumor volume reduction was 32.3 % at dose of 40 mg/kg (*p* < 0.05) and 64.5 % at dose of 80 mg/kg (*p* < 0.01), respectively. The tumors were excised and weighed at the end of week 10. A dose-dependent decrease of tumor weigh was observed in honokiol-treated groups (Fig. 7b). The tumor weight of 80 mg/kg honokiol-treated group was decreased by almost 90 % comparing to the control group (*p* < 0.01). The changes of body weight were measured weekly after honokiol treatment. No significant difference between control and honokiol-treated groups was observed throughout the experimental protocol (Fig. 7c). Besides, neither visible sign of toxicity nor any abnormal behavior were observed in honokiol-treated mice.

### Honokiol decreases the PCNA and CD31 levels in the tissue of SAS SP xenograft tumor

**Fig. 8:**
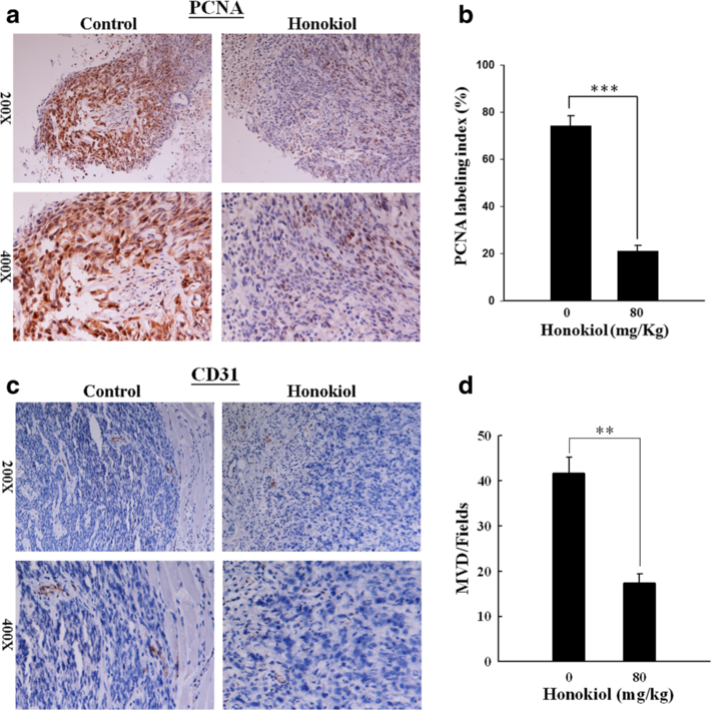
Honokiol markedly decreases the immunohistochemical staining of PCNA and CD31 in SAS SP xenograft tumor tissue. Immunohistochemistry staining of PCNA and CD31 was performed in the paraffin-embedded tissue sections of tumors from mice treated with or without honokiol (80 mg/kg). **a** The staining intensity (*brown color*) of PCNA was markedly lower in honokiol-treated group. **b** The PCNA labeling indexes of control and honokiol-treated groups. **c** The staining intensity of CD31 (endothelial cell marker) was markedly lower in honokiol-treated group. **d** The number of CD31 positive microvessel was counted at 200x magnification under a microscope. Significantly reduced microvessel density (MVD)/fields was observed in honokiol-treated group. Data shown are mean ± SD. **p < 0.01; ***p < 0.001, significant difference vs. control

The immunohistochemical examination was performed in the sections of excised tumors with or without 80 mg/kg honokiol treatment. In accordance with the reduced tumor volume and weight, the honokiol-treated tumors displayed lower PCNA (proliferating cell nuclear antigen) positive rate (Fig. 8a). The PCNA labeling index in control group was reduced from 74.13 ± 4.1 to 20.87 ± 2.4 (*p* < 0.001) by honokiol (Fig. 8b). Similar result was also observed in the staining of angiogenic marker, CD31 (Fig. 8c). As shown in Fig. 8d, the number of CD31-positive microvessels (MVD/fields) was significantly reduced from 42.7 ± 3.5 to 17.3 ± 2.1 by treatment with honokiol (*p* < 0.01), indicating that honokiol may inhibit neovascularization within tumor tissues of the SAS SP xenograft.

## Discussion

The resistance of OSCC to conventional chemotherapy or radiation therapy might be due to existence of CSCs [37]. Consequently, agents capable of eliminating this CSC population are desirable for improving the clinical outcomes of OSCC treatments. Many preclinical studies had shown the anticancer activities of honokiol [24]. Recently, our group and Ponnurangam et al., had reported the elimination of CSC-like population by honokiol in OSCC and colon cancer cells through Wnt/β-catenin [20] and notch pathway inhibition [27], respectively. This study now further demonstrated its inhibitory effects on the survival/proliferation signaling such as JAK2/STAT3, AKT, and ERK in the CSC-like SAS sphere cells and confirmed the in vivo effectiveness in xenograft animal model.

Generally, SP has been proposed as a practical method to enrich and isolate CSCs from many tumor tissues and cell lines [14]. Several studies had demonstrated that SP isolated from OSCC cell lines indeed possesses the properties of CSCs and higher tumorigenicity [16–18]. However, Broadley et al. had shown controversial results that the SP isolated from glioblastoma multiforme cells did not have enhanced stem-like property and tumor initiating activity over the non-SP cells, suggesting that the CSCs enriched by SP technique should be further confirmed by animal experiment [38]. In our results, the SP percentage in OECM-1 (20.5 %) is much higher than that in SAS (2.9 %) cells. This phenomenon is in accordance with the report by Chiou et al. that OECM-1 expressed higher ABCG2 compared to SAS cells [33]. However, the SAS cells are much more tumorigenic and metastatic than the OECM-1 cells [34]. Considering this controversy, we performed an animal experiment to confirm that the SAS SP did have much higher tumorigenicity (approximately ten thousand times higher) than the non-SP. Therefore, we used SAS SP xenograft as a model to evaluate the effectiveness of honokiol.

The effects of honokiol on the increase of Bax to Bcl-2 ratio and subsequent apoptosis induction had been reported in various types of cancer cells [39]. The significance of Bax to Bcl-2 ratio on the progression of several diseases or malignant tumors had been investigated by several studies [40]. This ratio may serve as a predictive marker to evaluate prognosis in patients with rectal carcinomas who have undergone elective colectomy and received post-surgery adjuvant treatment [41]. Our results further demonstrated the increased Bax to Bcl-2 ratio in the CSC-like SAS sphere cells after treatment with honokiol, indicating the potential of honokiol to improve OSCC therapy via apoptosis induction of CSCs. Compared to OECM-1 spheres, the honokiol-induced late apoptosis was more dominant in SAS sphere cells, suggesting the application of honokiol in the high-grade and aggressive OSCC might be more useful. Further clinical investigation is warranted.

Honokiol had been shown to induce apoptosis in various types of cancer cells through inhibition of several well-known survival/proliferation signaling pathways such as JAK/STAT, PI3K/Akt and MEK/Erk [42–45]. As these pathways also govern the CSC maintenance and survival [28–31], the honokiol-mediated inhibition of these pathways and apoptosis induction in CSC-like sphere cells would provide further mechanisms underlying its CSCs elimination potential.

The STAT3 signaling also mediates IL-6 induced EMT (epithelial-mesenchymal transition) to promote the metastasis of head and neck tumor cells [46]. The inhibitory effect of honokiol on EMT by targeting STAT3 signaling was recently reported [47]. In line with this, we also observed inhibitory effects of honokiol on the migration of SAS cells induced by IL-6 and on the STAT3 activity in SAS sphere cells. Furthermore, the contribution of STAT3-mediated EMT on CSC-like phenotype had also been noted [48, 49]. It is possibile that honokiol also suppressed the STAT3-EMT-promoted CSC-like traits in the microenvironment within the xenograft tumor. Further investigation is needed.

Constitutive activation of the STAT3 is associated with not only cell proliferation and metastasis but also angiogenesis [50, 51]. It is known that anti-angiogenesis via STAT3 inactivation also plays an important role in the honokiol-mediated anticancer activities [52]. In agreement with this, our immunohistochemical results show that not only PCNA but also CD31 (endothelial marker) were markedly suppressed in honokiol-treated xenograft tumor tissues, indicating that honokiol may be regarded as a useful antiangiogenic agent for the treatment of OSCC.

## Conclusions

In conclusion, our results have demonstrated that honokiol may induce apoptosis and inhibit the survival/proliferation signaling pathways in oral CSC-like cells. These effects were associated with the suppressed sphere formation in vitro and the reduced neovascularization and growth in xenograft tumors. The clinical development of honokiol as a novel complementary and alternative therapeutics targeting for CSCs to improve the clinical outcome of OSCC is warranted.

### Availability of data and materials

The effects of Honokiol (5 μM) and siSTAT3 on the cell viablity of SAS cells in the 24-h wound healing assay are provided as supplementary information in Additional file 1: Figure S1.

## Additional file

Additional file 1: Figure S1. Honokiol (5 μM) and siSTAT3 did not affect the cell viability of SAS cells in the 24-h wound healing assay. (A) SAS cells were treated with honokiol (5 μM) for 24 h in the same culture condition shown in Fig. 6b for wound healing assay. The cell viability was then determined by the quantitative staining of cellular proteins by sulforhodamine B. (B) After transfection with siSTAT3 and analysis for the expression of STAT3, the SAS cells were seeded into 6-well plate in the same condition shown in Fig. 6b for wound healing assay. After 24 h of incubation, the cell viability was then determined by the quantitative staining of cellular proteins by sulforhodamine B. (TIFF 1240 kb)

## Abbreviations

ABC: ATP-binding cassette
CSCs: cancer stem cells
EMT: epithelial-mesenchymal transition
MVD: microvessel density
OSCC: oral squamous cell carcinoma
PCNA: proliferating cell nuclear antigen
PI: propidium iodide
SP: side population
