# Supplementary figures and images for: Honokiol inhibits sphere formation and xenograft growth of oral cancer side population cells accompanied with JAK/STAT signaling pathway suppression and apoptosis induction
Source: BMC Cancer. 2016 Mar 24;16:245. doi: 10.1186/s12885-016-2265-6 (PMC4806492; doi:10.1186/s12885-016-2265-6)

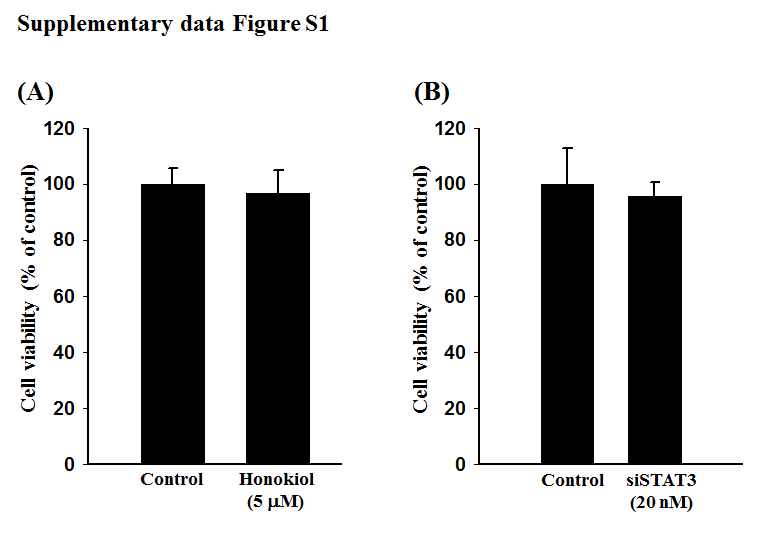

Supplement: Additional file 1: Figure S1. — Honokiol (5 μM) and siSTAT3 did not affect the cell viability of SAS cells in the 24-h wound healing assay. (A) SAS cells were treated with honokiol (5 μM) for 24 h in the same culture condition shown in Fig. 6b for wound healing assay. The cell viability was then determined by the quantitative staining of cellular proteins by sulforhodamine B. (B) After transfection with siSTAT3 and analysis for the expression of STAT3, the SAS cells were seeded into 6-well plate in the same condition shown in Fig. 6b for wound healing assay. After 24 h of incubation, the cell viability was then determined by the quantitative staining of cellular proteins by sulforhodamine B. (TIFF 1240 kb) [file 12885_2016_2265_MOESM1_ESM.tiff]
